# Higher risk, lower reimbursement: medicare payment paradox in cranial neurosurgery

**DOI:** 10.1007/s10143-025-04025-8

**Published:** 2026-02-04

**Authors:** Daniel Schneider, Ethan D. L. Brown, Timothy G. White, Daniel G. Eichberg, Aladine A. Elsamadicy, Daniel M. Sciubba, Sheng-Fu Larry Lo, Jung Park

**Affiliations:** 1 Department of Neurological Surgery, The Ohio State University Wexner Medical Center, Columbus, OH USA; 2 Department of Neurosurgery, North Shore University Hospital, Manhasset, NY USA; 3 Northwell, New Hyde Park, NY USA

## Abstract

Background and purpose To quantify variations in Medicare reimbursement for cranial neurosurgical procedures from 2014 to 2022 and evaluate associations with sociodemographic, clinical, and geographic factors. Methods This retrospective cross-sectional regression analysis of state-level Medicare data reviewed cranial surgery-related DRGs (21–30, 61–63, 81–83, 113–115, 131–133). We used multivariable linear regressions with heteroscedasticity-robust standard errors to assess associations between standardized per capita Medicare payments and variables including age, gender, race/ethnicity, dual-eligibility status, risk category, procedure volume, and region. Results were reported using unstandardized and standardized coefficients across 459 state-year observations. ResultsMean cranial DRG payment was $25,234. The Northeast and West had significantly higher payments than the South (β = $3,952 and $3,781, respectively; *P* <.001). Each SD increase in the percentage of Black and other non-White beneficiaries was associated with $957 and $875 higher payments, respectively (*P* <.001). Dual-eligibility rates predicted higher payments (β = $1,300 per SD; *P* <.001). In contrast, high-risk status was associated with lower payments (β = –$1,616 vs. low-risk; *P* =.001). Payments demonstrated a consistent upward trend of $905 annually (*P* <.001), with older populations showing higher payments (β=$728 per SD, *p* =.002). Conclusions Medicare payments for cranial neurosurgery varied by region, race, socioeconomic status, and risk. High-risk patients received notably lower reimbursement, raising concerns about potential disincentives for treating complex cases. These findings highlight opportunities to improve risk adjustment and promote equitable neurosurgical reimbursement.

## Introduction

Cranial procedures represent a significant component of Medicare expenditures within the neurosurgical domain, encompassing operations for conditions ranging from traumatic brain injury to cerebrovascular disease and intracranial neoplasms [1]. As healthcare costs continue to rise and policymakers seek to optimize resource allocation, understanding the factors that influence Medicare payments for these procedures has become increasingly important [2]. Previous research has documented substantial geographic variations in Medicare spending across various service categories [3], yet the specific determinants of payment variation for cranial procedures remain incompletely understood.

Medicare payment structures are designed to account for clinical complexity through diagnosis-related group (DRG) classification, but questions persist regarding whether current adjustment mechanisms adequately address sociodemographic factors that may influence resource utilization and costs [4]. Disparities in neurosurgical care access and outcomes have been well-documented across racial, socioeconomic, and geographic dimensions [5]. However, less attention has been paid to how these factors may relate to payment variations within the Medicare system.

The intersection of payment policy with healthcare disparities has particular relevance for cranial procedures, which often involve complex, resource-intensive care for vulnerable populations [6]. Recent healthcare policy reforms have emphasized value-based reimbursement and equitable care delivery, yet implementing these principles requires nuanced understanding of current payment patterns and their determinants [7]. 

Despite growing literature on healthcare payment variations, few studies have comprehensively examined the sociodemographic, clinical, and geographic factors that influence Medicare payments specifically for cranial procedures [8]. This gap limits our ability to assess whether current payment structures appropriately accommodate patient and population characteristics that may drive resource utilization and costs.

Our study addresses this knowledge gap by analyzing Medicare fee-for-service payments for cranial DRGs across the United States from 2014 to 2022. Using multivariable regression analysis, we examined the associations between payments and key factors including beneficiary demographics, socioeconomic status, risk category, discharge volume, and geographic region. This approach allows us to disentangle the relative contributions of these factors to payment variation while controlling for potential confounders.

By identifying the patterns and determinants of Medicare payment variation for cranial procedures, our findings provide insights that can inform policy discussions around payment equity, resource allocation, and healthcare disparities. These insights have particular relevance as Medicare continues to evolve its payment methodologies to better align reimbursement with care complexity, quality, and value.

## Materials and methods

### Data sources and study population

We utilized data from the Medicare Geographic Variation Public Use File (2014–2022) and the Medicare Inpatient Hospitals - by Geography and Service datasets to examine Medicare fee-for-service (FFS) payments. The study focused on cranial-related inpatient services using predefined diagnosis-related groups (DRGs): 21–30, 61–63, 81–83, 113–115, and 131–133. These DRGs encompass procedures including craniotomy, ventricular shunt, intracranial vascular procedures, cranial nerve operations, and cranial/facial trauma treatments. For each state and year, the average Medicare payment for cranial-related DRGs was calculated by averaging the DRG-specific payments across all the predefined DRG codes. As a retrospective study using a public, anonymized database, this study was exempt from IRB review and did not require participant consent.

### Study design

This retrospective cohort study analyzed the association between sociodemographic factors and Medicare payments for cranial-related inpatient services across the United States from 2014 to 2022 importantly capturing both pre-pandemic and post-pandemic reimbursement patterns. The outcome variable was the standardized per capita Medicare payment for cranial-related DRGs, which removes geographic payment rate variations and adjusts for population differences.

Independent demographic variables included mean beneficiary age (centered around the overall mean), proportion of female beneficiaries (dichotomized as above or below median), and racial composition (percentage of Black, Hispanic, and other non-White beneficiaries). Independent socioeconomic factors included percentage of beneficiaries with dual Medicare-Medicaid eligibility. Independent clinical variables included risk score (categorized into tertiles to create three risk groups: Low, Medium, and High). Independent healthcare utilization variables included total DRG discharges. Independent geographic variables included state where the procedure was conducted (categorized into geographic regions based on the United States Census Bureau’s standard classification of Northeast, Midwest, South, and West). Delaware, Maryland, and the District of Columbia were grouped into the South instead of North to reflect Medicare Administrative Regions and hospital referral networks, where these states are often grouped with Virginia and other Southern states for health care policy. Finally independent temporal variable included procedure year (centered around 2018).

### Statistical analysis

A multivariable linear regression model was employed to assess the impact of independent variables on Medicare payments for cranial-related DRGs. To address potential multicollinearity, we calculated Variance Inflation Factor (VIF) scores with a threshold of 5. CMS Hierarchical Condition Categories risk scores were categorized into three levels (low, medium, high), and continuous variables, such as age and year, were centered. States were grouped into regions to improve model parsimony and interpretability. This study utilized complete case analysis, with state-year observations containing incomplete data excluded from regression models.

Linearity between independent variables and the dependent variable was assessed using scatter plots. We used heteroscedasticity-robust standard errors (HC3) to account for potential heteroscedasticity, following best practices for finite small samples [9, 10]. The normality of residuals was tested using the Shapiro-Wilk test and visual inspection of Q-Q plots. The Durbin-Watson test was performed to check for autocorrelation in residuals. To validate our findings, we performed two sensitivity analyses: (1) replacing categorical risk tertiles with the continuous beneficiary risk score to ensure binning did not distort relationships, and (2) reassigning Delaware, Maryland, and the District of Columbia to the Northeast region to test the robustness of geographic disparities.

For percentage variables (racial composition and dual eligibility), we report standardized coefficients (per standard deviation increase) to facilitate comparison of relative impact. For binary variables (risk levels, gender proportion, and regions), we report unstandardized coefficients. Statistical significance was determined at *P* <.05. All analyses were conducted using Python (version 3.9) with the statsmodels package (version 0.13.2).

## Results

We analyzed 459 observations of cranial DRG payments across the study period. The mean Medicare payment was $25,233.80 (SD, $5318.30) with a range of $15,750.10 to $46,989.30. The mean percentage of beneficiaries with dual eligibility was 19.1% (SD, 6.3%). Regarding racial composition, Black beneficiaries comprised 8.1% (SD, 10.1%), Hispanic beneficiaries 4.0% (SD, 4.9%), and other non-White beneficiaries 6.2% (SD, 8.5%) of the study population. The mean number of total discharges was 1844.2 (SD, 1866.2), ranging from 17 to 10,114 discharges. Risk stratification classified 25.3% of observations as high-risk and 38.1% as medium-risk. Regional distribution included 17.6% from the Northeast, 25.5% from the West, 23.5% from the Midwest, and 33.4% from the South.

The multivariable regression model significantly predicted Medicare payments for cranial DRGs (F = 63.71; *P* <.001), explaining 55.1% of the variance (adjusted R² = 0.538). Table 1 presents the complete regression results. High-risk status was associated with significantly lower Medicare payments compared with low-risk status (coefficient = -$1616.47; 95% CI, -$2592.16 to -$640.79; *P* =.001), while medium-risk status showed no significant difference (coefficient = -$91.32; 95% CI, -$781.42 to $598.78; *P* =.795).

**Table 1:** Multivariable regression results for predictors of medicare payments for cranial DRGs

Predictor	Coefficient (95% CI)^a^	*P* Value
Risk Category^b^		
High vs. Low	-$1616.47 (-$2592.16 to -$640.79)	0.001
Medium vs. Low	-$91.32 (-$781.42 to $598.78)	0.795
Demographic Factors		
Age (per SD)^c^	$727.53 ($258.33 to $1196.72)	0.002
Female proportion above median	$234.31 (-$386.49 to $855.10)	0.459
Race and Socioeconomic Factors (per SD)		
Black beneficiaries percentage	$957.38 ($563.03 to $1351.74)	< 0.001
Hispanic beneficiaries percentage	$29.93 (-$289.00 to $348.86)	0.854
Other non-White beneficiaries percentage	$874.66 ($417.92 to $1331.40)	< 0.001
Dual eligibility percentage	$1299.60 ($941.07 to $1658.14)	< 0.001
Other Factors		
Total discharges (per discharge)	$0.19 ($0.03 to $0.35)	0.019
Year (per year from mean)^d^	$905.35 ($778.16 to $1032.53)	< 0.001
Region (vs. South)		
Northeast	$3952.06 ($3042.64 to $4861.47)	< 0.001
West	$3781.00 ($2674.88 to $4887.13)	< 0.001
Midwest	$743.11 ($130.68 to $1355.54)	0.017

^a^Coefficients represent US dollars. SD indicates standard deviation; CI, confidence interval. The model adjusted for all variables shown. Model R² = 0.551; adjusted R² = 0.538; F = 63.71; *P* <.001

^b^Risk categories are compared with low risk (reference). Regional comparisons use South as the reference category

^c^Age was centered around the mean

^d^Year was centered around 2018

Each standard deviation increase in beneficiary age was associated with a $727.53 increase in payments (95% CI, $258.33 to $1196.72; *P* =.002). Female gender proportion above the median did not significantly affect payments (coefficient = $234.31; 95% CI, -$386.49 to $855.10; *P* =.459).

A one standard deviation increase in dual eligibility percentage was associated with a $1299.60 increase in payments (95% CI, $941.07 to $1658.14; *P* <.001). For racial composition, each standard deviation increase in percentage of Black beneficiaries (coefficient = $957.38; 95% CI, $563.03 to $1351.74; *P* <.001) and other non-White beneficiaries (coefficient = $874.66; 95% CI, $417.92 to $1331.40; *P* <.001) was associated with higher payments, while the percentage of Hispanic beneficiaries showed no significant association (coefficient = $29.93; 95% CI, -$289.00 to $348.86; *P* =.854).

Total discharges showed a modest but significant positive association with payments (coefficient = $0.19 per additional discharge; 95% CI, $0.03 to $0.35; *P* =.019). Medicare payments increased by $905.35 for each year from the centered mean (95% CI, $778.16 to $1032.53; *P* <.001).

**Fig. 1:**
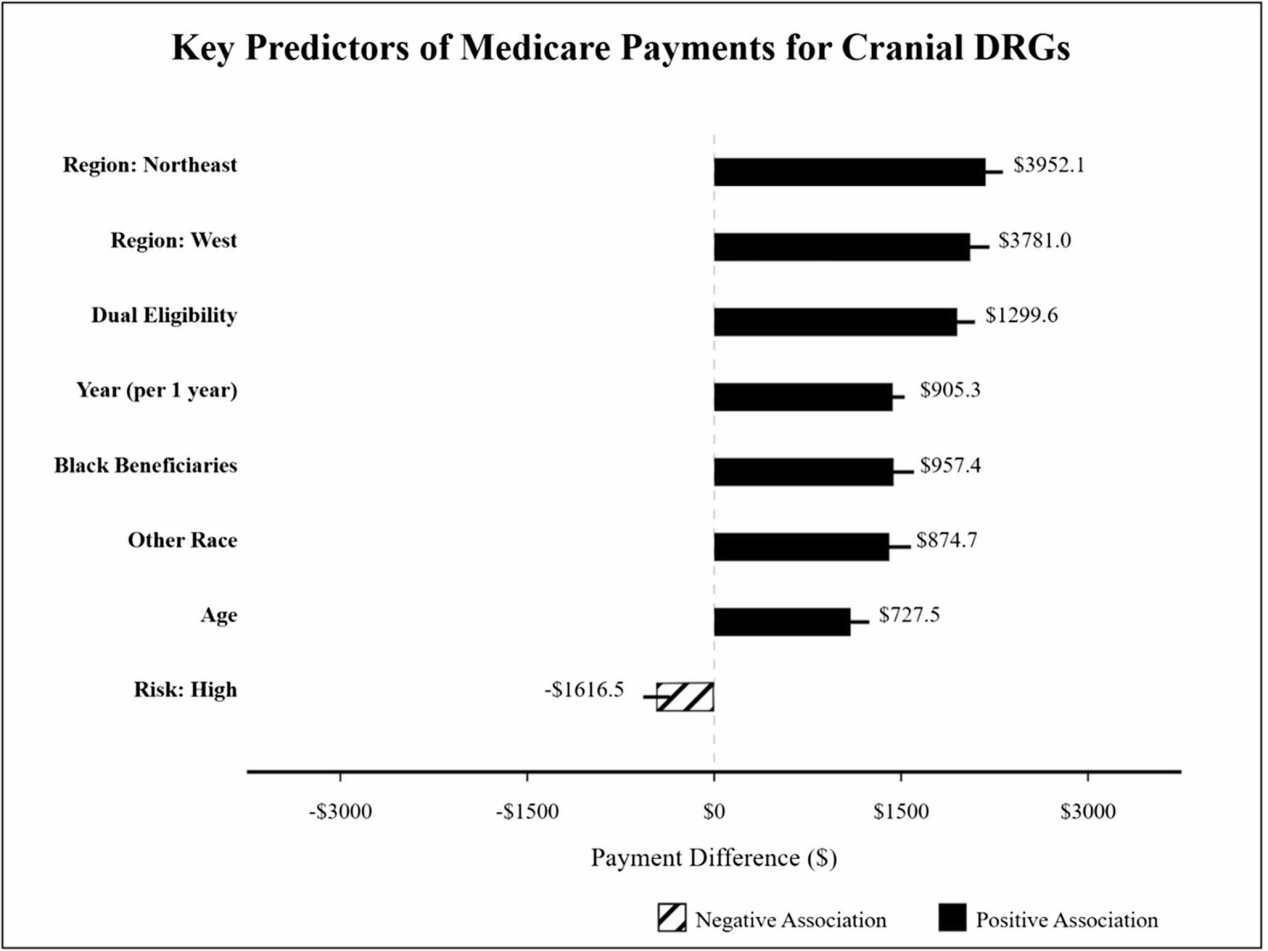
Key Predictors of Medicare Payments for Cranial DRGs. Regression coefficients for predictors of Medicare payments for cranial diagnosis-related groups (DRGs) are shown. Bars represent the estimated payment difference associated with each predictor, with horizontal lines indicating 95% confidence intervals. For continuous variables (age, race percentages, and dual eligibility), coefficients represent the effect of a 1-standard deviation increase. For risk categories and regions, coefficients represent the difference compared with the reference category (low risk and South region, respectively). Solid black bars indicate positive associations; striped bars indicate negative associations. All displayed predictors are statistically significant (*P* <.05) except where noted in Table 1

Significant regional variations were observed, with the Northeast (coefficient = $3952.06; 95% CI, $3042.64 to $4861.47; *P* <.001) and West (coefficient = $3781.00; 95% CI, $2674.88 to $4887.13; *P* <.001) regions showing substantially higher payments compared with the South. The Midwest region also demonstrated higher payments than the South (coefficient = $743.11; 95% CI, $130.68 to $1355.54; *P* =.017). Figure 1 illustrates the relative magnitude and direction of these key predictors.

### Model robustness

The Durbin-Watson statistic was 2.173, indicating no significant autocorrelation in residuals. The condition number was large (7.9 × 10⁴ for the regular model and 1.8 × 10⁴ for the standardized model). Residual analysis revealed non-normality (Jarque-Bera test, *P* <.001) with positive skew (2.144) and high kurtosis (11.004).

Sensitivity analyses also confirmed the robustness of the primary model. When replacing categorical risk groups with a continuous average beneficiary risk score, the inverse relationship between risk and payment persisted (*P* <.001), suggesting that the observed risk-payment relationship was not an artifact of variable categorization. Additionally, reclassifying Delaware, Maryland, and the District of Columbia from the South to the Northeast region yielded consistent geographic trends, with the Northeast (coefficient = $5769.61; 95% CI $4774.84 to $6764.38; *P* <.001), West (coefficient = $4,596.90; $3467.39 to $5726.43; *P* <.001), and Midwest (coefficient = $1669.63; 95% CI $1135.655 to $2203.61; *P* <.001) continuing to receive significantly higher payments compared to the South.

## Discussion

Our analysis of Medicare payments for cranial DRGs reveals significant variations associated with geographic, socioeconomic, racial, and clinical factors. These findings contribute to a growing body of literature on healthcare payment disparities and regional variation in Medicare spending [1–3]. 

The substantial regional differences in cranial DRG payments, with the Northeast and West receiving significantly higher payments than the South, align with established geographic variations in overall Medicare spending [3]. However, our analysis demonstrates that these regional disparities persist even after adjusting for demographic characteristics, risk profiles, and socioeconomic factors. This suggests that payment variations may be driven by regional practice patterns, differential resource utilization, or variations in reimbursement methodologies rather than solely by population differences [5]. The magnitude of these regional differences, nearly $4000 higher per admission in the Northeast compared with the South, represents a substantial financial impact given the approximately 1800 average annual discharges in our dataset.

Our findings regarding the association between dual eligibility status and higher Medicare payments merit particular attention. The strong positive relationship between dual eligibility percentage and payments ($1300 increase per standard deviation) indicates that areas serving higher proportions of socioeconomically disadvantaged beneficiaries receive greater Medicare reimbursement for cranial procedures. This contrasts with some previous research suggesting that hospitals serving disadvantaged populations may receive lower payments due to case-mix adjustment inadequacies or quality penalties [11]. Our findings may reflect the complex needs of dual-eligible beneficiaries, who often present with multiple comorbidities and require more resource-intensive care during hospitalization [12]. 

The racial disparity patterns observed are particularly noteworthy. Higher percentages of Black and other non-White beneficiaries were associated with significantly higher Medicare payments, while Hispanic beneficiary percentage showed no significant relationship. These racial associations persisted despite controlling for dual eligibility and risk categories. Previous research has documented racial disparities in neurological care access and outcomes [13, 14], but our findings suggest a more complex picture regarding payment structures. The higher payments associated with areas serving larger proportions of Black and other minority beneficiaries may reflect greater disease severity, higher complication rates, or differences in procedure types within DRG categories. These results emphasize the importance of examining racial disparities through multiple lenses, including cost and resource utilization patterns.

The counterintuitive finding that high-risk status was associated with lower payments (-$1616 compared with low-risk) challenges conventional assumptions about payment models. This inverse relationship may reflect several potential mechanisms: high-risk patients might receive less aggressive interventions, experience shorter lengths of stay due to higher mortality, or encounter systematic underpayment relative to their actual care needs [15]. 

This finding represents perhaps the most concerning aspect of our results, as it suggests a potential two-tiered system of neurosurgical care. An economic disincentive for treating high-risk Medicare patients could manifest clinically in treatment decisions that prioritize less resource-intensive procedures. For example, instead of performing a complex resection for a perirolandic tumor requiring awake craniotomy with neurophysiological monitoring, surgeons might opt for a simpler needle biopsy, a less optimal but more economically viable approach given the reimbursement constraints.

Such practice pattern shifts, if occurring systematically, would constitute a troubling disparity where the complexity of neurosurgical care becomes determined by insurance status rather than clinical necessity. The payment differential represents a substantial financial pressure that could influence institutional and physician decision-making, particularly for facilities operating with narrow margins or serving predominantly Medicare populations.

These results highlight a need to reevaluate risk-adjustment methodologies for neurosurgical DRGs to ensure that payment structures properly incentivize appropriate care for all patients regardless of risk status. Without addressing this inverse risk-payment relationship, facilities serving higher proportions of complex patients may face unsustainable financial pressures, potentially exacerbating access barriers for the most vulnerable populations.

The temporal trend in cranial DRG payments ($905 annual increase) far exceeds general healthcare inflation rates during the study period [16]. The modest but significant volume-payment relationship ($0.19 per additional discharge) provides some evidence for economies of scale in cranial procedures, though the effect size suggests these economies may be limited. A more likely context involves broader changes in Medicare payment policy, which have created a widening divergence between facility and professional reimbursement [17]. Although inflation-adjusted Medicare reimbursement for neurosurgical professional services has declined by approximately 33% over the last two decades, as described by Haglin et al., our findings corroborate a steady increase in total episode payments [17]. This decoupling reflects the structural difference between physician fees (Part B) and hospital payments (Part A), which accrue annual inflationary adjustments via the IPPS Market Basket. This trend was corroborated in a 2025 systematic review by Hopkins et al., who reported that while neurosurgical professional pay remained stagnant, nonprofit hospital revenue and executive compensation increased by mean values of 199% and 709%, respectively, since 2011 [18]. Thus, rising DRGs observed in our study may reflect facility-side inflationary updates rather than an increase in the valuation of surgical work, presenting several implications for the regionalization of complex neurosurgical care and hospital consolidation trends [17]. 

The inverse relationship between high-risk status and payments raises critical concerns about the adequacy of current payment structures for complex neurosurgical cases and suggests a potential systemic problem where financial disincentives could inadvertently lead to less optimal surgical interventions for the highest-risk patients. This finding, coupled with the substantial annual increase in payments over time, suggests ongoing evolution in cranial procedure reimbursement that merits closer examination.

Our results have several potential policy implications. First, the substantial regional payment variations suggest the need for greater standardization in Medicare reimbursement methodologies across geographic regions to ensure equitable resource allocation. Second, the counterintuitive relationship between risk status and payments warrants reevaluation of risk-adjustment mechanisms for neurosurgical DRGs to prevent potential underpayment for high-risk patients. Third, the associations between racial composition and payments highlight the need for more nuanced approaches to addressing disparities that consider not only access and outcomes but also resource utilization and payment adequacy.

For clinical practice, our findings underscore the financial complexities of providing cranial neurosurgical care to diverse Medicare populations. The inverse relationship between high-risk status and payments raises concerns around the adequacy of current payment structures for complex neurosurgical cases and suggests that financial disincentives could inadvertently lead to less optimal surgical interventions for the highest-risk patients. This finding, coupled with the substantial annual increase in payments over time, suggests an ongoing evolution in cranial procedure reimbursement that merits closer examination. Neurosurgical providers and hospital administrators should be aware of potential payment inadequacies for high-risk patients and consider these financial dynamics when developing service lines and care pathways.

## Future directions

Future research should examine these relationships at more granular levels, including hospital-specific and patient-level analyses that can better account for clinical complexity and procedural variations. Incorporating clinical outcomes data would allow examination of the value proposition of whether higher payments translate to better patient outcomes across different demographic groups and regions.

Detailed clinical investigation into treatment decision-making for high-risk neurosurgical patients, particularly comparing procedure selection across different payment systems, would be valuable to determine whether the financial disincentives identified in our analysis translate to differences in clinical practice. Such research could examine whether high-risk Medicare patients receive different surgical approaches (e.g., biopsy vs. resection for complex tumors) compared to similarly situated patients with different insurance coverage.

Longitudinal analyses tracking how recent policy changes, including value-based purchasing initiatives and alternative payment models, affect these payment patterns would provide valuable insights for policy refinement. Additionally, comparative analyses across different procedure types could determine whether the sociodemographic associations observed for cranial procedures represent broader patterns in Medicare reimbursement.

As healthcare payment systems continue to evolve, understanding these complex relationships between patient characteristics, geography, and payments will be essential for developing equitable reimbursement policies that support high-quality neurosurgical care for all Medicare beneficiaries.

## Limitations

Our study has several important limitations. First, we used state-level aggregated data rather than individual patient or hospital-level information, which may mask facility-specific variations in payments and practice patterns. This ecological approach limits our ability to make inferences about individual patient factors driving payment differences. Second, DRGs may encompass heterogeneous procedures with varying levels of complexity and resource requirements. While stratifying DRGs by specific procedure codes or comorbidities could have provided greater granularity, we were limited by privacy constraints inherent to the Medicare Geographic Variation PUF. As CMS suppresses data for geographic units with fewer than 11 discharges, increasing the granularity of our query was not feasible due to potential for non-random missing data across low population states. Without procedure-specific codes or clinical details, we were unable to determine whether payment variations reflected differences in procedure mix, complication rates, or other clinical factors within DRG categories.

While our analysis identifies significant associations, it cannot establish causal relationships between sociodemographic factors and Medicare payments, as unobserved confounding variables may explain some of the observed associations. Moreover, we observed a high condition number suggesting multicollinearity among demographic predictors and residual analysis demonstrating non-normality. However, VIF remained within acceptable limits and our use of HC3 robust standard errors ensured the OLS estimator remained valid despite these distributional deviations.

## Conclusions

Our findings provide important insights into the factors influencing Medicare payments for cranial procedures. Geographic location emerged as the strongest predictor of payment variation, with substantially higher payments in the Northeast and West compared with the South, even after adjusting for demographic and clinical factors. Socioeconomic status, as measured by dual eligibility percentage, showed a strong positive association with payments, challenging assumptions that facilities serving disadvantaged populations necessarily receive lower reimbursement. The racial composition of beneficiaries demonstrated nuanced relationships with payments, with higher percentages of Black and other non-White beneficiaries associated with increased payments. Collectively, these results highlight the complex interplay between demographic, socioeconomic, and geographic factors in determining Medicare payments for cranial procedures. The substantial explained variance in our model indicates that these factors represent important determinants of payment variation.

## Data Availability

No datasets were generated or analysed during the current study.
